# Dr. Monro on Medullary Sarcoma of the Stomach and Intestines

**Published:** 1830-01-01

**Authors:** 


### XXXV.

### Medullary Sarcoma of the Stomach and Pharynx.*

The obscurity of abdominal tumours and diseases is proverbial. Every day an enlarged liver is mistaken for an ovary, or diseased ovary for a large liver— ascites and ovarian dropsy are constantly confounded — morbid growths of the kidney are overlooked — affections of the spleen discovered on the dissecting-table — and malignant diseases of the stomach either never found out at all, or not till they become so marked and unequivocal that none who have eyes in their head can neglect to observe them. These are not hypothetical blunders, or mistakes got up for the sake of an antithesis, for we have ourselves seen examples of each and all within the last year and a half. Such being the case we cannot pay too much attention

* Monro on the Morbid Anatomy of the Gullet, Stomach, and Intestines. 2d Edition.

to well-authenticated cases of organic disease of the abdominal viscera, at least if it is our object to avoid cutting a sorry figure in the eyes of patients and well-informed practitioners.

Fungus hæmatodes or medullary sarcoma of the stomach is, we think, more obscure in its symptoms and march than scirrhus of that organ. Dr. Monro, who has lately published the second edition of his valuable work on the Diseases of the Intestinal Canal, informs us in his chapter on the subject, that he has met with several instances of this disease. In two there were tumours of a somewhat similar description in the substance, and also on the surface of the liver, which was very much enlarged; the lymphatic glands in the vicinity were also considerably enlarged and indurated. If we might judge from our own experience we would be inclined to say that the liver was of all the internal organs most frequently affected secondarily with fungus hæmatodes. But this by the way. Our author subjoins the history of those cases which are contained in the Anatomical Museum of the University of Edinburgh.

*Case* 1. "In this there is, at the cardiac extremity of the stomach, an extensive lobulated tumour of a medullo-sarcomatous nature, the lobes of which are covered with a delicate mucous membrane. In the right lobe of the liver there are two *scirrhous* tubercles. The left lobe adheres to the cardiac extremity of the stomach; and at the attached extremity, there is a tumour, the centre of which is occupied by a sloughy, fungoid mass, extending through an aperture into the stomach. Symptoms: Countenance exsanguine, tenderness in epigastrium, vomiting, alternate constipation and diarrhœa, and emaciation. Patient a female, aged forty-six."

It there be no looseness in the foregoing statement and the liver really presented true scirrhous tubercles whilst the stomach was affected with medullary sarcoma, the case is possessed of additional interest as shewing the connexion between the two forms of malignant disease. We have seen a preparation in the possession of Mr. Brodie shewing the co-existence of cauliflower excrescence of the uterus, scirrhus, and fungus hæmatodes in the same individual, and it really is not unfrequent to find tumours undistinguishably resembling the fleshy tubercle of the uterus along with fungus hæmatodes of another part. Many able surgeons are inclined at the present day to consider scirrhus and fungus as merely grades of the same disease.

*Case* 2. "At the cardiac extremity of the stomach there is a large medullary fungoid tumour, of a light straw colour, covered by a delicate vascular membrane. The serous membrane of the diaphragm was much thickened, and intimately adhered to the left lobe of the liver, and to the stomach. The patient, a man of seventy-three years of age, had a sense of constriction in the throat, combined with difficult deglutition, which was relieved by the use of the probang. He also had cough, constipation, latterly diarrhœa, and became much emaciated."

*Case* 3. A man, ætatis 44, complains of pain in the right hypochondriac and epigastric regions, much increased by pressure which produces nausea and retching — considerable hardness and swelling in the upper part of the abdomen, tension and elasticity in the lower —immediately under the false ribs of the right side, two hard tumours distinctly felt, each apparently about the size and shape of a walnut—appetite impaired—breathing difficult—cough—head-ache sometimes severe — much thirst—pulse natural—tongue white—bowels slow—urine scanty. Says that about five weeks ago he felt frequent sharp shooting pains in the epigastrium, which were followed by the gradual enlargement of the parts.

Opening medicine, a sinapism to the epigastrium, and leeches were applied with relief to the pain, but on the next night the feet and ankles became œdematous. The emaciation increased, the pain returned with severity, the lower extremities became more swollen, fluctuation was evident in the abdomen, and the patient died on the eighteenth day.

"On opening the abdomen, the liver appeared much increased in size. A number of large tuberculated tumours were found on its surface. Some of these, when cut into, were of a hard cheesy consistence, while others, more advanced, were in a state of suppuration. The abomen contained about lb. xv. of serum."

It is to be remarked that although the patient in this case complained of nausea and retching, it was after pressure on the epigastrium, and not, if we may trust the silence of the reporter, after eating or drinking. This, of course, is not the ordinary character of malignant disease of the stomach itself, and so far may assist in diagnosis. Besides there was dropsy, another concomitant of hepatic, as distinguished from gastric disease.

"It may not be improper to add, that medullary sarcoma of the stomach is not always similar to the medullary matter of the brain, being sometimes more of a yellow, reddish or brown colour, and also that there is some variety as to the consistence of different parts of the tumour; the consistence towards the circumference being harder than that in the centre of the tumour, which may perhaps be owing to an imperfect suppuration going on in that part of the swelling, as, according to Mr. ABERNETHY, happens when the primary disease is seated in the testicle.

"There is also a coincidence between the external medullary sarcoma, and the internal, which grows from the mucous membrane; in respect that in both there is swelling and enlargement of the glands connected with the absorbent system.

"In one of the specimens in the Museum, the mucous membrane of the stomach is ulcerated, and there were melanotic depositions on it; and there is a large irregular opening in the stomach communicating with the centre of a medullo-sarcomatous tumour of considerable size, which occupied the whole of the smaller curvature of the stomach.

"Medullo-sarcomatous tumours, of a harder description, sometimes grow from the mucous membranes. I have in my possession a specimen of this description, which grew from the pharynx, and proved fatal, by obstructing respiration and deglutition."

The following interesting case was received by Dr. Monro from his late pupil, Dr. George Wylie, of Paisley. It is rather out of place in a section professedly devoted to fungus hæmatodes of the *stomach*, but n'importe. Valuable facts are still valuable, although they may owe nothing to the arrangement and method of their narrator.

*Case.* Roseanne Grey, æt. 11, of ruddy complexion, with dark hair and eyes, was much exposed to cold during the earlier part of the spring of 1810, whilst sitting at work, and constantly complained of a chilliness. About the end of March she complained of a pain in the head, and especially in the left ear, accompanied by giddiness, which were much increased by exercise. She became weak, and of unhealthy appearance, and for three weeks in April was confined to bed. During the summer months, she was now and then so free from her complaints, for a few days at a time, as to be able to stir abroad. About the middle of August her relations first observed that she spoke less distinctly.

"About the end of September I first saw her. She complained of pain in the head and left ear, and giddiness; her deglutition and speech were impaired; the mouth was a little distorted to the right; she breathed none through the left nostril; heard less distinctly with the left ear; and the eye of that side was so full that the eyelids could not completely cover it, yet she saw equally well with them both. She was much emaciated; had little appetite, and was costive; pulse small, frequent, and weak. On looking into the mouth, a tumour was observed to be situated partly behind, and to the inside, of the backmost grinder on the left side of the upper jaw; its base extended to the gum before, and to the velum palati behind, stretching to the right as far as the suture which joins the palate bones. It was about the size of a small nutmeg, and had a groove or depression alongst its middle from before backward. When pressed it was slightly elastic, and had so much the appearance of an abscess as to be repeatedly mistaken for one. It was a little moveable; and when moved, gave a sensation similar to that felt from moving a small hard steatoma."

Laxatives were prescribed with benefit to the general health, and Dr. Wylie saw no more of the patient till the 13th of November, when the tumour had increased rapidly, was as large as a pippin, immoveable, and filling the fauces so completely that liquids were swallowed with great difficulty.

"It was connected with the gum of the backmost grinders and the tonsil of both sides, and completely hid the velum and uvula. It was larger on the left side, and, from its great projection, depressed the root of the tongue, so as to give the appearance externally of a tumour above the pomum Adami. By depressing the tongue with a spatula, the uvula was seen to be pushed toward the right, and depending from the posterior part of the tumour. The right half of the tumour was firm, the larger or left half was a little elastic.

"On the 16th, a small oblong superficial ulcer appeared on the fore part of the tumour; a second was in a few days formed by the pressure of the under molaris of the left side; neither of these gave any pain, and they discharged very little; the first healed in a short time. More than the usual quantity of saliva was secreted; and. from the difficulty of deglutition, it almost constantly ran from the mouth.

"On the 20th the tumour was observed to be larger; and the patient breathed and swallowed with extreme difficulty. Inspiration became more difficult in the horizontal posture, especially during sleep, and she was frequently awaked suddenly by it, and had recourse to the semi-erect posture, like one in a paroxysm of asthma. In a consultation, it was agreed that extirpation should be speedily resorted to, for the following reasons: The tumour, though fixed, was at first moveable; from its rapid increase the patient must in a short time have died from inanition or strangulation; the operation would at the least produce a temporary alleviation: And a case occurred lately, in which a similar tumour was removed with success.

"21st, Betwixt the hours of one and two p. m. the tumour was removed by incision, after being freed from its attachments to the gum and tonsil on the right, and the cheek, gum, and tonsil on the left side. The velum and uvula were removed along with it, which exposed to view a tumour nearly as large as the first; they were connected at their bases, and seemed to be only one tumour with two lobes. This posterior lobe hung, from its attachment behind the velum, down into the pharynx. The effusion of blood from the wound was trifling; the pulse 120, and weak. Feeling weak and much exhausted, she was put to bed, with the intention of waiting till she became stronger before any thing further should be attempted.

"The tumour being cut into, was found to be of the sarcomatous kind; very like gristly fat in texture, but of a rather whiter colour."

In the course of about an hour hæmorrhage occurred to a considerable amount but was arrested. On the two next days she vomited dark grumous matters, the result, no doubt, of the blood which she had swallowed. With regard to the wound it went on so well that on the 29th it was clean and free from fetor, and the patient was able to walk about the house without support. On the 3d of December, however, the face, especially the left side, was much swelled and occasionally flushed, the tumour behind the velum had increased much, and externally a small, conical tumour, hard and immoveable, was observed projecting below the mastoid process of the left temporal bone. She began to hear less distinctly, was frequently seized with faintness, and was confined to bed. After a few days the tumours grew rapidly, respiration and deglutition became proportionally more difficult, she dozed constantly unless when tormented with pain, but awoke almost suffocated, and on the 27th of January, 1811, death put a period to the poor young creature's sufferings.

" *Dissection.* — By sawing through the inferior maxilla at the middle, and turning back the left half, after dividing its muscular connexions, the tumour was fully exposed to view. It now appeared to fill the whole of the throat and pharynx. Its base was attached to the palatal plates of the palate bones, to the left of the pterygoid process of the sphenoid, and the point of the petrous portion of the temporal bone, where the eustachian tube issues. Its left side grew to the cheek, the gum, and tonsils, and sent a sharp process to occupy the space betwixt the angle of the jaw and the mastoid process. Its posterior surface was connected by cellular membrane, to the back of the pharynx. As it passed the nostrils, it sent a process into each ; that into the left was bloody. Upon opening the cranium, the base of the tumour was observed to have obliterated the extremity of the os petrosum and corresponding portion of the sphenoid bone, causing the dura mater over the cavernous sinus to be elevated as high as the clinoid processes. The left nervus trigeminus could not be traced after it pierced the dura mater. The hole which the tumour had made in the bones was roundish, with sharp and rough edges ; and was large enough to admit the point of the forefinger. The tumour seemed to have no connexion with the orbit. About an ounce of water was found in the ventricles of the brain.

" The tumour, at its attachments, was firm and almost cartilaginous ; but its apex, which rested on the gullet, was soft and friable. The processes which were in the nostrils were as soft as brain."

This case exemplifies well the bad effects of the operation for fungus hæmatodes after ulceration has commenced. A quicker growth, more destructive ravages, and more extensive implication of other organs appear to be the result of an operation under such circumstances. It seems as if the system deeply resented the interference of art and the sequel forms the most apt illustration of that saying — God never made his work for man to mend!

To revert more particularly to the present case we regret that no mention is made of the state of other parts, but that all the dissector's attention has been taken up in describing the local mischief. One word with regard to the operation performed : — no effectual precautions would appear to have been taken to guard against hæmorrhage, which *was* alarming and *might* have been fatal. Surgeons, we fear, too generally imagine that provided the immediate bleeding is stopped, all that remains is to wash their hands and faces, and put the patient into bed. They have been led of late years to consider it unnecessary to tie any vessel short of the aorta, and at length, we suppose, they will tie none at all. The best surgeon, in our humble opinion, is he who provides not only for what is present, but for what may come ; who sits down leisurely to secure those vessels which will probably bleed when the patient is warm in bed, or flushed with the fever which must certainly succeed. In removing tumours about the jaw or the fauces, the cautery, actual or potential, is of great importance, as it acts not only in providing against hæmorrhage, but in completing or aiding the operation of the knife.

---

